# Book Reviews

**Published:** 1812-01

**Authors:** 


### CRITICAL ANALYSIS

### OF RECENT PUBLICATIONS

IN THE

### DIFFERENT BRANCHES OF PHYSIC, SURGERY, AND MEDICAL PHILOSOPHY.

*An Inquiry into the Process of Nature in repairing Injuries of the Intestines, illustrating the Treatment of penetrating Wounds and strangulated Hernia. By* BENJAMIN TRAVERS, Demonstrator of Anatomy, Surgeon to the Hon. East India Company, and to the London Infirmary for curing Diseases of the Eye. 8vo. p. 384. London. Longman and Co. 1812.

THE only fair and correct way to ascertain how far the merit of originality belongs to a professional writer, is to compare his opinions with those which were previously current among the best informed men of his time. If they were unknown to the most learned of the profession, and did not occur to them on occasions which call forth their utmost reflection and knowledge of the subject, we may fairly view them in the light of discoveries, and may safely assert, that their being known in former times detracts no more from the merit of the writer, than it does from the practical value of the opinion. In perusing old and neglected works, and poring over the multiplicity of forgotten doctrines which they contain, we are struck by those chiefly whose truth we ourselves had previously observed. In most instances in which an author publishes unknown but previously-recorded opinions, it is not the record which led to the discovery of the truth, but the discovery of the truth which led to the notice of the record. A re-discovery is almost the only means of restoring forgotten knowledge, for, if the living generation are thoroughly ignorant of it, it matters little whether it is buried in unexplored nature, or in neglected books under cobwebs and dust.

We intend to employ these principles for the purpose of appreciating the benefit which the present author has conferred on his profession. We must first, however, lead our readers through the inquiry, and present them with as just a view of it as can be seen through the contracted limits of our publication.

Wounds of the intestine are of two kinds; the one simple, when the parietes of the abdomen remain entire; the other complicated, when the same cause which wounded the bowel, wounded wounded also the parietes: these two forms of injury, it is obvious, bear the same relation to each other, as simple and compound fractures.

When an intestine is wounded, there are several circumstances which tend to prevent its alimentary contents being extravasated into the abdomen; thus effusion is much less likely to occur if the alimentary canal is empty, than if it is full, at the time of the accident; much too will depend not only on the absolute size of the wound, but on its relative magnitude to the bowel in which it is inflicted. The same sized aperture in the colon is less likely to be followed by effusion, than if it had occurred in the jejunum, because in the former instance a larger portion of canal remains entire for the uninterrupted passage of the fœces. Another cause which tends to prevent effusion is the completeness with which the viscera of the abdomen fill up the space. We are in the habit of talking of the cavity of the abdomen; the fact, however, is, that there is no cavity, as is readily observed in the opening of bodies; for no sooner does the knife penetrate the peritoneum, but the gut starts out from the wound; every point in the surface of the intestines is in the closest possible contact either with the surface of other intestines, of other viscera, or of the peritoneum which lines the parietes of the abdomen. Nature has packed them with the utmost closeness, which, among other uses, serves to prevent the wounded bowel from spilling its contents into the abdomen. A wound across the intestine is less likely to admit of extravasation than one along the course of the canal; the circular muscular fibres being more powerful than the longitudinal, and drawing the edges of a longitudinal wound asunder, so as to convert a slit into a circular aperture. If the aperture is an incised wound, it will speedily be followed by adhesion, and other changes, which tend to its obliteration, and to the consequent prevention of effusion; but, if it is the result of ulceration, these changes, and the consequence, are less likely to occur. If the wound in the bowel is followed by a discharge of blood, or air into the abdomen exterior to the gut, effusion of fœces is more likely to occur; because fluid or air offers less resistance to the discharge of the contents of the intestine, than the viscera or the parietes of the abdomen. These are the principal circumstances which seem to influence the effusion of the fœces into the abdomen, in wounds of the intestines; and this influence is so considerable, that in a number of these cases (as our readers may learn by reference to the experiments, cases, and quotations, which are given at length in Mr. Travers's book) extravasation is not the consequence; when it occurs it is inevitably fatal, whereas wounds completely through the abdomen, which, when we consider the way in which it is filled, must inevitably have been conjoined with wounds of the bowels, are sometimes followed by such trifling symptoms as to have led some of our elder writers to conclude that the bowels were not injured. Unless the gut is full, and the wound extensive, effusion rarely happens. Extravasation is much less likely to happen when the parietes of the abdomen are wounded at the same time with the bowel, than when an aperture is caused in the latter, while the former remains entire. This sometimes happens from a violent blow which ruptures the bowel, or from a sharp instrument lodged within its cavity, or from an ulcer which destroys its structure, and occasions an orifice in the abdomen: of these latter, a sufficient number of cases is given by Mr. Travers, to show that they are by no means rare occurrences.

The professed object of Mr. Travers's inquiry is to get a more minute and accurate insight, than has hitherto been attained, into the process by which unassisted Nature endeavours to repair injuries of the bowels. This mode of investigation admits of an infinitely more advantageous application, in those cases which belong to the surgeon, than to those which belong to the physician; to the cure of a wound, than to that of a fever. Solution of continuity, in all their varieties of kind, are restored by a process thoroughly natural; and, as the office of the surgeon is in these cases that of a mere assistant to nature, it is obviously wise to learn what he has to aid, before he offers his assistance. With this view, Mr. Travers has prosecuted and detailed a long course of experiments, all of which consist in wounding the bowels; and when the consequent process of nature has concluded, whether in death or in recovery, in ascertaining, by careful dissection, what that process has been.

"If a gut (says Mr. T.) be *punctured*, the elasticity of its peritoneal coat, and the contraction of its muscular fibres, open the wound, and the villous or mucous coat forms a sort of hernial protrusion which obliterates the aperture. If an incised wound be made, the edges are drawn asunder, and reverted so that the mucous coat is elevated in the form of a fleshy hip. If this section be transverse, the hip is broad and bulbous, and acquires tumefaction and redness from the contraction of the circular fibres behind it, which produces relatively to the everted portion the appearance of a cervix. If the incision is according to the length of the cylinder, the hip is narrow, and the contraction of the adjacent longitudinal fibres, resisting that of the circular, gives the orifice an oval form. The eversion and contraction is produced by that series of motions which constitute the peristaltic action of the intestines."

The principal means, however, by which nature achieves the restoration of a wounded bowel, is the adhesive inflammation to which the peritoneum, like all the membranes lining closed cavities, most providentially has a natural aptitude. When a bowel and the parietes abdominis are wounded, without being followed by fœcal effusion into the belly, (for in this case the injury is inevitably fatal,) the adhesive inflammation is liable to be excited by two causes; one is the solution of continuity in the structure of the intestine itself; the other is the displacement of a mass of intestine from its natural situation, and from the living surfaces with which it is naturally in contact, which occurs when the bowel is protruded through the wounds in the integument.

"In the abdomen (says Mr. T.) the moveable as well as the fixed viscera preserve a certain relative position, adapted to the performance of their several functions. In a prolapse of the bowel, through an opening of the parietes, the protruded folds undergo a change of relative position greater or less according to the extent of the protrusion. If the intestine be gradually and softly returned in its natural order, it recovers position and function without much consequent inflammation. If, on the other hand, it be put back in the mass without attention to the course of the canal, its organic function becomes suspended and it becomes an irritant; inflammation rapidly supervenes, lymph is effused in flakes, and the folds are universally agglutinated."

It becomes, therefore, a most important object to restore protruded intestines in their natural order; for, in proportion to the displacement is the degree of diffused inflammation, and in proportion to that inflammation is the danger. Experiment E. affords a complete illustration of what Mr. Hunter meant when he said it was "not necessary that both surfaces which are to be united should be in a state of inflammation for the purpose of effecting an union; it appears only necessary that one should be in such a state which is to furnish the materials, viz. to throw out the coagulating lymph, and the opposite *uninflamed* surface accepts simply of the union."

"An incision (says Mr. Travers) was made in the belly of a dog, so as to expose a portion of the small intestine, in which an opening was made that admitted the large end of a silver blow-pipe. A piece of omentum protruding with the gut was cut away. The latter was softly replaced within the wound, which was now closed by suture. In a week he was perfectly recovered from the consequent symptoms. I then killed and examined him. The injured fold had receded from the wound, though it was connected by a little band of lymph to the peritoneum at that part. On laying open the bowel, the aperture was discovered of its original dimensions, but completely covered by the lateral adherence of the sound fold which lay nearer to the abdominal muscles. A probe passed into it, took an oblique course between the two folds, under a delicate stratum of lymph which connected them."

Such cases, however, are illustrations rather than proofs of the truth of Mr. Hunter's doctrine; because, although the surface of intestine, which adhered to the wound of its neighbour, neighbour, was itself sound and unwounded, there is no proof that it was not inflamed; the contrary was most probable, considering the circumstances of the experiment, the causes of inflammation which attended it, and the natural aptitude of the peritoneum to be thrown into the adhesive inflammation.

To the experiment quoted above, and to several cases and experiments of a similar tendency, Mr. T. observes,

> "That further essential advantages are derived from the absolute fullness of the abdomen; that it not only prevents effusion, but provides the means of restoration; that the process of spontaneous reparation essentially depends upon the indefinite extension of the peritoneum, (by which membranous surfaces, identical in their organisation and properties, are every-where opposed,) and upon its disposition to assume the adhesive inflammation. Thus, if a bowel be wounded lying in contact with the peritoneum of the muscles, it repairs itself by the mutual adhesion of the cut surfaces; another more distant from the parietes contracts a close adhesion with the contiguous fold, or lays hold of the adjacent omentum. If the wound is an orifice from loss of substance, it is never obliterated by the deposition of new matter, but by the permanent close adhesion of the surface applied to its margin: if, on the contrary, it is simply a division of substance, as in a clean cut wound, the sides may unite *per se*, without contracting any surrounding adhesions."

When an intestine receives a transverse wound so as completely to cut across the canal as far as the mesentery, the accident is invariably fatal, and is generally followed by extravasation of fœces into the abdomen. In some of Mr. Travers's experiments, however, notwithstanding the extent of the wound, extravasation was completely prevented, partly by the orifices being considerably diminished in magnitude by the contraction of the circular fibres around them, and the protrusion of the villous coat, and partly by a plug of chylous matter detained at the orifices. A complete transverse wound was inflicted on the intestine of a dog which had fasted seven hours. On examination after death, nothing like intestinal matters appeared in the abdomen; "the ends of the gut, concealed by surrounding adhesions, were separated, contracted, and everted, to the utmost; and both orifices were totally obliterated. Each section presented a continuous smooth surface, the apertures being sealed by a yellowish substance, much resembling curd or lymph. This proved, on closer examination, to be a portion of chylous matter, so firmly condensed in the orifice, that a stream of water injected did not dislodge it. The diameter of the orifices, when the plug was washed away, did not exceed the tenth of an inch."

In such cases, however, even although effusion may be thus prevented, the accident will most probably be fatal; because the continuity of the canal is destroyed by the retraction of the orifices to a distance from each other, and the only possible or imaginable chance of safety is in the neighbouring intestines, or omentum, forming adhesions around the edges of the orifices so as to produce a cylinder from one orifice to the other, and thus afford a substitute for that portion of the natural canal which had been lost by the retraction of the cut extremities. This curious example of natural substitution has actually sometimes occurred. "Shipton, an English student of surgery in the early part of the last century, made the following experiment. Having cut away a portion equal to two fingers' breadth of the ilion of a dog, he connected the extremities by an uninterrupted suture, and closed the external wound. The cicatrices being completed, and the parts examined at the end of three weeks, it was found that the sewed intestine lay at a considerable distance from the wound, firmly attached to the peritoneum, but the suture had yielded so as to allow the cut ends of the gut to recede, which were enveloped in a sac formed of adhering omentum and intestine;" and this sac must have served to convey the aliment past the wounded portion of the canal, for the dog was not killed till three weeks after the experiment. Mr. Travers also made a similar experiment with similar result.

"The small intestine of a dog, who had been kept without food, was exposed, and half of its cylinder was cut across. It was carefully replaced and the wound closed; he recovered from the immediate symptoms, but died on the ninth day following, from circumstances unconnected with the experiment, of which I was not previously aware. On examination, the peritoneal surface was found to be healthy, and the wounded intestine thus curiously enveloped. A pouch, resembling somewhat the diverticulum in these animals, was formed opposite to the external wound, on the side of the parietes, by the living peritoneum; on the other side by the mesentery of the injured intestine, that intestine itself, and an adjacent fold which had contracted with it a close adhesion. The pouch thus formed and insulated, included the opposed sections of the gut, and had received its contents, viz. a ball of hair which the animal had licked from the wound, bone, and other solid substances. The sides of the sac were smeared with fœcal matter. The tube at the orifices was narrowed by the half eversion, but offered no impediment to the passage of fluids."

The result of this inquiry, therefore, as far as we have hitherto proceeded in it, is, that the means by which unassisted Nature attempts the permanent restoration of wounded intestines, is the adhesion of the wound either to its own edges, or to the membranous surfaces which are contiguous to it.

In wounds of the abdomen which penetrate into the intestines, if the patient is not speedily destroyed by hæmorrhage, which is the case if the aorta, or cava, or any other very large vessel, or very vascular organ, as the liver, are injured, injured; and, if no effusion takes place into the abdomen, which Mr. T. has produced abundant proofs is by no means a common consequence of these accidents, the principal danger then is that of diffused peritoneal inflammation.

"It will be observed in the examination of the records (says he) that, while one class of cases presented so little derangement of the natural and the vital functions, as to induce the belief that the bowels had escaped uninjured; in another class the symptoms were of such urgency and danger as to render the patient's recovery unexpected and surprising. Now, by whatever difference in the circumstances of the injuries, or the constitutions of the patients, these opposite results may be explained, the inflammation of the peritoneal surface in the one class, and its absence in the other, is, I doubt not, the real cause of the distinction. Constitutional disposition we know influences, in a very sensible degree, the character of a local inflammation in all parts of the body. This continuous inflammation is in some cases erysipelatous, and tending directly to gangrene; but, when arising from local injury in a healthy subject, more commonly assumes a phlegmonous character, and proves fatal in the adhesive stage.

"The remedies (for the peritoneal inflammation) on which I feel disposed to rely, are few and simple. Total abstinence from solid food, and a drink, easy of absorption, given in very sparing quantities, and at long intervals, with a rigid preservation of the supine posture, are restrictions which should be most rigorously observed as long as any are indicated."

The necessity of abstinence, for the purpose of keeping the wounded intestine empty, was strikingly illustrated by one of the author's experiments. The intestine of a dog was cut half across the calibre, and the wound was closed. He vomited once or twice, but next day seemed recovering. In the course of the day, however, he drank several ounces of water, became worse towards the evening, and died two days from the beginning of the experiment. The peritoneum was found inflamed, and a considerable quantity of water, undoubtedly that which he had drank, was effused into the abdomen.

When a wound penetrates through the parietes of the abdomen into an intestine, and the fœces are discharged externally, if the patient be properly treated, that is, if a low spare diet be rigidly adhered to, and blood-letting be employed according to symptoms, the common consequence is, that the injured bowel adheres to the aperture in the parietes, and, although the fœces continue for some time to be discharged externally, after a few weeks they begin to pass by the natural channel, and cease to appear at the external wound, which fills up, skins over, and becomes permanently obliterated. As long as the injured portion of the bowel continues incapable of transmitting the fœces, they are discharged at the external wound; but, as soon as it has had time to receive a restoration of its organisation, and its functions, functions, it reassumes its office; and the wound of the parietes, being no longer prevented from healing by frequent interruption from the fœcal discharge, closes of itself. Mr. Travers gives a number of cases of this description from the Medical Commentaries, Dessault's Journal, the writings of Luis, Memoirs of the Academy of Surgery at Paris, Mr. Hunter, and M. Larrey. In cases such as these, surgeons, fearful that the discharge at the wound may become permanent, and degenerate into an artificial anus, have often made attempts to hasten its closure: these attempts, if they have in any degree succeeded, have generally been followed by symptoms of returning inflammation of the bowel, by vomiting, pain, and fever, which are only relieved by a free feculent discharge at the wound. The fact is, not that the fœces resume their natural channel because the wound closes, but the wound closes because the fœces have returned to their natural channel. Surgeons have mistaken the order in which the parts of the process succeed each other.

"I need scarcely say (says Mr. T.) that, in substituting the natural for the artificial anus, it is necessary to proceed with the most delicate caution. It is common for the more solid part of the food to lodge and be detained in the renovated portion of the canal. There is a tendency to accumulation wherever an extensive wound has been inflicted upon the intestine, first from the contraction, proportioned to its extent, which has been shewn to take place at the moment of the injury; secondly, from the contraction which accompanies the healing process; thirdly, from the impaired action of that part of the gut which has been the subject of the lesion. The discharge per anum should therefore be attentively observed, both as to its consistence and quality: if it cease, or ceases to correspond with the diminished evacuation at the wound, under the continued use of injection, the belly will become tender, and the febrile symptoms be re-excited. Under such circumstances, the exhibition of mild laxatives by the mouth may prove advantageous; but, should the constipation be obstinate, and the abdominal pain and tension continue, the wound must without hesitation be dilated, and the bowel be again unloaded."

Evacuation from the intestinal canal is one of the principal means of obviating or removing the symptoms of inflammation which are liable to occur; and, as long as we are unable to effect this per anum, we must effect it by the wound. This is a maxim of the utmost importance in the treatment of this class of injuries. When the fœcal discharge becomes permanent at the wound in the integuments, it forms what has been called the artificial anus. The external discharge, in all cases, continues until the bowel has resumed its organisation and functions; and, if any thing should occur to produce in it a permanent disability, the consequence will be a permanent discharge at the wound. Thus, "a surgeon, having removed a portion of wounded gut equal to a hand's breadth in extent, with the view of sewing together the sound parts of the tube, accidentally let slip the lower portion, which receded into the belly; the upper was then stitched to the wound, and thus was established a permanent artificial anus." It is, however, not always so readily explained. Mr. Travers advocates the opinion of Dessault, that it arises from the cut ends of the bowel adhering to the wound of the parietes, so as to form with each other so acute an angle that the fœces cannot readily turn round it. It is the strongest possible argument for sewing the intestine in cases of wounded bowel complicated with prolapse, because, under this treatment, the cut edges of the intestine adhere to themselves, without forming any adhesion to the parietes of the abdomen, and thus is prevented all chance of that angularity, or that contraction of the wounded portion of bowel, which is so far capable of impeding the passage of fœces along the natural channel as to cause their permanent discharge at the external wound.

Wounded bowel with prolapse is more dangerous than without, because of the greater degree of inflammation which attends the former kind of injury.

"If (says Mr. T.) the prolapsed intestine be reduced in such a manner that each part may recover its natural situation, a vigilant after-treatment will generally subdue inflammatory symptoms. If the bowel be returned hastily and in mass, the patient will rarely, if ever, escape a fatal inflammation. The scrupulous directions which we have received from systematic writers to return the protruded intestine inch by inch in the order of its proximity to the wound, or inversely to the order of its descent, are founded in more soundness of observation than their readers have generally apprehended."

In these cases there are two modes of practice which have been employed by surgeons: the one is to stitch the bowel before returning it, the other is to return it without stitching: both kinds of practice have often terminated successfully, as will appear from the cases detailed in the work; but Mr. Travers prefers the former, for the following reasons:

"The grand objections to the practice of returning a wounded bowel without a suture, are the heavy drain upon the system if the evacuation be alimentary; the irritation occasioned by the continual discharge, and the tardiness of the healing process; the danger of future impediment to the free course of the matters from a permanent angularity of the adhering fold, or the incroachment of the parietes on the tube in healing; and lastly, of future prolapse, and even artificial anus, from the actual deficiency of the parietes intestinalis corresponding to the extent of the cicatrix. The objections now stated do not lie against the suture. The intestine being directly reduced recovers position and function, the cylinder is perfect of itself and not formed by the walls of the abdomen. If the cases recorded by practical writers be compared, it will be found that the objections stated above to the spontaneous cure are not exaggerated."

To ascertain the comparative merits of different ligatures on on the wounded bowel, Mr. T. made a number of experiments. The intestine of a dog was cut across, and a single ligature was employed to keep the cut edges together, opposite the mesentery ; the consequence was, that the intervening spaces between the mesentery and the ligature which remained unsecured, gaped into two round mouths, and effusion and death followed. Three single stitches were found to be liable to the same objection in a less degree. So strong is the action of the circular muscular fibres of the intestine, that it is not easy to confine together the edges of a longitudinal wound. Mr. Astley Cooper, however, succeeded in effecting it, in some experiments on dogs. Mr. Travers found that the most effectual way to heal the wound with the nearest possible approximation of its edges, was by a neat continued suture. "A small sewing needle and a silk ligature were employed." Most of our readers we suppose are already aware that Dr. Thompson, by some well-contrived experiments, ascertained, when a wounded bowel was secured by a ligature on the cut edges, that the ligature, during the healing process, passed inwardly, and was deposited in the canal of the intestine. This is the mode which Nature provides for the safe expulsion of the ligature. Wherever, therefore, this means becomes necessary to secure a wounded intestine, it is obvious that the ends ought to be cut off before the bowel is returned, for, if the ends of the ligature are left hanging out of the wound, we provide the most effectual means that could be devised to counteract this salutary provision of nature.

Dr. Thompson's experiments had already sufficiently shown, that, when a ligature was put on a wounded intestine, it always passed inwardly ; but one of Mr. Travers's experiments has illustrated this in a manner still more unexpected and wonderful. "A ligature of thin packthread was firmly tied round the duodenum of a dog, so as completely to obstruct it. The ends of the string were cut off, the bowel returned, and the abdominal wound was then closed." On the 15th day, having, most probably to the surprise of the experimentalist, passed stools, and completely recovered from the symptoms which the operation had at first produced, the dog was killed, and examined. "The folds of the omentum contiguous to the intestine, round which the ligature had been passed, adhered to it at several points. A slight circumferential depression was observed in the duodenum. The gut was then carefully laid open. The villous surface was more vascular, and of a deeper colour, than usual. A transverse fissure only marked the seat of the ligature."

"The

"The inflammation (says Mr. T.) which a ligature induces on either side of it, is terminated by a deposition of a coat of lymph exterior to the ligature, which quickly becomes organised. When the ligature thus inclosed is liberated by the ulcerative process, it falls of necessity into the canal, and passes off with its contents; 'the fissures left by the ligatures are gradually healed up, but the opposed villous surfaces, so far as my observation goes, neither adhere nor become consolidated by granulation, so that the interstice marking the division internally is probably never obliterated.' Ligatures of every description unconfined at the external wound separate into the canal; not from any law in the economy which has been adduced in explanation of some similar invariable phenomena of which the cause was not obvious, but from their speedy and complete investment by the uniting medium."

Having now presented our readers with a very full, and we hope a clear, insight into the first half of Mr. Travers' Inquiry, we for the present lay it aside. The foregoing details, as will appear in the sequel, have a very near relation to the pathology and treatment of strangulated hernia, but we must defer showing the particulars of this relation until a future number of our Journal.

---

*Some Observations upon Diseases, chiefly as they occur in Sicily. By* William Irvine, M.D. F.R.S. Ed. of the Royal College of Physicians, London, and Physician to his Majesty's Forces. 8vo. pp. 120. Murray, London.

In presenting an account of a work which treats of diseases in a climate we have never visited, we can offer little more than a few select passages from it, and pronounce a general opinion of its merit. In his capacity of physician to the forces, the intelligent author had abundant opportunities of acquiring experience, and his private practice we are informed was extensive. His work throughout, affords proof of quick perception, and a clearness of mind which allows a man to state what he actually witnesses, without perverting it by preconceived notions or deep-rooted prejudices.

The volume is divided into twelve chapters, which treat of the following subjects: 1. On the climate of Sicily. 2. Of the continued fevers of Sicily. 3. Of summer fevers. 4. Of autumnal fevers. 5. Of winter fevers. 6. Of the possible advantage in other climates of the practice recommended in the former chapter. 7. Of intermittents and remittents. 8. Of the dysentery of Sicily. 9. Of phthisis. 10. On hepatitis. 11. Of rheumatism. 12. Of the medical use of borax, with some observations on erysipelas.

"The Island of Sicily lies between 36° 25′ and 38° 25′ of North latitude, and between 12° 5′ and 16° 5′ of East longitude. Its climate is seldom very cold in winter, while in summer it is always oppressively hot. Till autumn little rain falls after the month of April; after the month of May almost none. Towards the end of August, or beginning of September, the rains begin: but the heat continues great till the middle of the latter month, after which it rapidly declines. In June and July fevers are frequent, and have much of an inflammatory type. In August and September they are still more common, and begin to be accompanied with more debility. From that time dysenteries increase in numbers; but after the end of October few new cases comparatively occur. From November till May again the heat is moderate, the thermometer seldom rising above 65 or 70, and often ranging as low as 50, and sometimes under that degree."

From the vicissitudes of heat being great, local inflammation is common in the winter, and phthisis pulmonalis is often fatal. This disease is supposed by the natives to be infectious, but Dr. Irvine could not obtain sufficient proof of this opinion being well founded. The symptoms of the complaint resembled those which characterise it in this country. In the treatment of it, digitalis was not beneficial; squills and opium, in large doses, with a frequent repetition of blisters, especially when employed in an early period of the disease, were very successful remedies.

Fevers are very prevalent in the island; the author has arranged them in three classes. To the first he refers those which occur during the hot months of summer; they have a very inflammatory type, run a short course, and bear with advantage plentiful evacuations.

"*Second Class.*—Towards the end of August, in September, in October, and sometimes later, another form of this disease appears, which is more rarely rapid in its course, which induces speedily a state of great debility that is not so certainly cured by general evacuations, and which is very frequently accompanied with universal yellowness of the skin."

From November to May fevers occur less frequently. "They are then more uniformly long in their duration, the inflammatory symptoms do not rise so high as in the first class, nor does debility come on so rapidly or violently as in the second. These, which form the third class, very much resemble the Synochus of England, and are not so fatal as either of the former classes."

In making this division the author wishes it to be understood that he does not assert there is any real difference in the nature of these fevers; on the contrary he is "disposed to join in opinion with those who consider fevers as divisible into intermittent and continued only; and that the latter, like the former, are variable in a thousand ways, by the constitution of the patient, his diet and manner of life, the heat, cold, dampness, and dryness, of the season or climate, certain ill-understood qualities of the air, and others not greatly more distinct of the soil."

The treatment which proved most salutary was repeated bleeding and cold affusion: a case of summer fever, with which we shall conclude, will best illustrate the author's judicious practice.

"Sept. 12, 1808.—H. S. ætatis 24, was seized three days ago with shivering, head-ach, and other symptoms of fever. Complains now of violent violent head-ach, giddiness, restlessness, and heat. Tongue covered with a thick white crust. Face flushed. Eyes suffused with blood. Pulse 120, full, but not strong. No stool to-day. Skin hot and dry.

℞. Magnesiæ vitriol. ℥j. statim sumend.

The cold affusion frequently.

"13.—Was extremely delirious all night. Several stools. In no respect better than yesterday. Eyes redder.

Continuatur affusio frigida.
Repet. magn. vitriol.
Mittatur sanguis ex arterio temporali ad ℥viij.
& repetatur vesperi si opus.
Adhibeatur vesicatorium capiti anteriori.

"14.—Lost fifteen ounces of blood. Much less delirious. Eyes better.

Repetatur arteriotomia.
Adhibeatur vesicatorium occipiti.

"15.—No delirium. Eyes almost well. Pulse 108, much stronger. Skin still hot, and look confused.

Repetatur arteriotomia ad ℥x.
Affusio frigida frequenter.
Magnesiæ vitriolatæ ℥j.

"16.—Nearly well. Slept a good deal. Tongue moistening.

Continuatur affusio.

"17.—No complaint. Excessively weak, and without appetite.

Continuatur affusio pro re nata.

℞. P. Jalap. gr. x. Calomel gr. v. Capiat secundo quoque die ad tertiam usque vicem.

"The patient required after this time no treatment. His appetite began to return in a few days, and then he had a little wine. He remained very weak, however, till about the middle of October."

---

### *Practical Observations on Cancer: by the late* John Howard, Surgeon Extraordinary to the Cancer Ward in the Middlesex Hospital. 8vo. Lond. 1811. pp. 114.

(Continued from Vol. 26, p. 504.)

THERE are so many obstructions to the early extirpation of tumours that are suspected to have a cancerous tendency, arising both out of the accidents of the case, and from the feelings of the patient, that every effort to elucidate the medical treatment of such cases is of value: a due sense of the utility of this part of the investigation will be an apology for our extracting rather largely from this portion of the work.

"As in diseased testis, so in the female breast, when the tumor changes from a quiet to an irritable state, something analogous to inflammation in the softer and external parts of its body takes place. This high degree of irritation proceeds not however to suppuration, but to a certain action or fermentation, and to extravasation, both within and without the gland; and sometimes to a slough, somewhat like the French "*suppuration gangréneuse*;" from a slough of the cellular membrane, in diseased habits, near the rectum; or, more properly, like the slough of a carbuncle. But there is more frequently a granulated kind of hardness in a diseased cancerous gland.

"As this stage advances, so is the hope of success from an operation more or less uncertain. When the disease has proceeded still further, and the axillary glands are affected, though the skin and integuments should be whole, such affection implies not only great irritation, but perhaps absorption of the cancerous fluid; but of this last I have some doubts. Be this as it may, from the great extent of the disease, success becomes more doubtful; and when the cancer affects parts adjoining, as in the case of diseased testis, where the complaint has spread from this gland to the chord, and to the glands of the corresponding kidney, nothing favorable can be expected ftom an operation. So in the female breast, when ulceration has taken place, with considerable surrounding hardness, even though the axillary glands may not be enlarged, the probability of relief from an operation is very feeble. And whatever opinion there may be with regard to the absorption of a diseased fluid before ulceration, and whilst the tumor is entire, I have no doubt of absorption taking place afterwards; one proof of which is the appearance of tumors in remote parts of the body, as well as in the neighbourhood of the ulceration; and another proof is the very rapidly colliquative symptoms attending the last most deplorable stage of the disease.

"Whatever means are employed to keep quiet a gland diseased from a blow or bruise, in cases where an operation will not be submitted to, the rule of practice of endeavouring to discuss it, or prevent its increase, at an *early period* holds good. From the time of the accident, it should never be neglected or left to itself. Applications of a sedative and discutient kind should be had recourse to immediately:—pressure from the stays should be guarded against:—the bowels should be kept open, and a cooling regimen enjoined; and it may be necessary to draw blood repeatedly, by means of leeches. It is before ulceration takes place, that leeches may be applied, at some distance from the injured part, and afterwards to the part itself.

"The action of discutients in promoting the absorption of blood extravasated from bruises is generally quick. If therefore the above means should fail, either to give ease or to remove any hardness that may remain, blisters may be repeatedly applied, which, by stimulating the skin and producing a considerable serous discharge, will probably prevent the injured part from taking on a diseased action. And where the injury has been neglected, and where, at a distant period, pain, enlargement, or preternatural fulness, has taken place, the like means may be had recourse to, *spatiis catameniorum intermediis*. And with the same precaution, a warm sea-bath, or where that cannot be had, an artificial sea-bath, or even the common warm-bath, may be employed. The immersion of the body draws the circulation and nervous power to the whole surface of the skin, and increases the sensible and insensible perspiration from the cutaneous glands and pores: it relaxes not only the injured parts, but the system in general, and thus I conceive relieves by causing a powerful resolution; but it is very probable that sea-water, as well as some mineral warm-baths, the Harrowgate for instance, may have a specific action, independent of their operation as warm water.

"Except when a stimulus is required, as in chlorosis, the diet in all cases where there is a cancerous tendency cannot be too strictly cooling. If it consisted wholly of vegetables, farinaceous substances, and milk, many lives might be saved, or at least prolonged; but, on the contrary, the majority of patients in this predicament have an unnatural appetite for luxurious eating,—and this exasperates the disease. Their digestion is faulty: they are subject to vomiting: the chylopoietic viscera do not perform their functions properly. If the bowels

4 are are particularly costive in one person, they are often irregular and lax in another, and from this laxity they seem to find relief. In the more costive, even drastic purgatives may be necessary; in the more delicate, when bilious affections prevail, Epsom-salt, or Kali Vitriolatum with Rhubarb and Magnesia, or Calomel, may be more proper.

"Much has been said and much expected of medicines of a SPECIFIC nature for the mitigation and cure of cancer; but this object, in my opinion, is not likely to be obtained by any one medicine, but by the co-operation of several, uniting to form and support different indications, according to the state, nature, and circumstances, of the various affections which go under the general name of cancer.

"EXCISION, if in the most early state of the disease, is a good remedy; but, as far as my experience has gone, I am almost led to believe that, *if external and internal means of relief are applied with due discrimination and judgment, sufficiently early, the knife even may be superseded.*—Where, from the established rules of surgery, as when a cancer is fixed, or adherent, this means cannot be employed, it is matter of curious inquiry to learn how many persons with cancers of this description go on for years, even to extreme old age, with only such a share of pain as can be submitted to, without much injury, yet performing all the common functions of life!—The fact is indisputable.

"I suspect, in these happy constitutions, nature, in some way not easily explained, works for their benefit, to which medicine has not hitherto sufficiently adverted. I know there are natural constitutions, though thoroughly cancerous, in which the disease proceeds slowly and without much irritation in its progress; and these are for the most part called cold habits, abstemious, and not given to idleness or to the luxuries of the table.—On the other hand, in full, more plethoric, obese habits and sanguinary temperaments, in those who indulge in wine, and good eating, without much air or exercise, the disease frequently goes on with rapidity.

"If these be facts, they point out the great necessity of a very abstemious regimen, with moderate exercise, in the alleviation and cure; and they show, amongst many other circumstances, that whatever irritates the part externally, or the system internally, must accelerate the disease,—and so we naturally find it. Hence, whatever the expectations of other medical men may be, as to a specific remedy for this disease, I am of opinion that it will never be found amongst the class of direct stimulants.

"But this requires a certain qualification: I believe the assertion is right, both with respect to internal and external stimulants, in the form of high diet, plaisters, caustic, &c.; but the operation of a blister, (though it be a strong irritant to the skin) has a concomitant which the common stimuli to the skin want, namely, *increased secretion.* —It is not the simple stimulus, but it is the combination of the two effects which makes blistering so remarkably useful.—This mode of relieving by blisters deserves to have been employed more frequently.

"In enlargement of glands in young women, the Cummin plaister was usually applied in the Middlesex Hospital, under the direction of the late Mr. Minors, with good effect; but medicines internally were at the same time employed. Here a blister would probably have been a better application. In a true cancerous tubercle, where there was considerable pain, I have used a blister without producing ulceration; and it had a temporary effect in relieving the pain, though it did not diminish the tumor. The tubercle to which it was applied, though painful, was not inflamed; but, as there were other tubercles in its neighbourhood, with tense skin and the purplish-blush, I was fearful fearful of going on with it. The excoriation healed, just as the excoriation of the skin does after this application. The woman lived a few years, without experiencing any ill effects from the blister; and she did not die of this disease at last.

"I am strongly inclined to believe that in all incipient, scirrhous, and cancerous, tumors, at an early period of life, after due relaxation by evacuation from the neighbouring vessels, by leeches, and in some instances by cupping, under a cooling diet, with the frequent use of purgatives, *much may be done by blistering the part repeatedly*,—having recourse to it in aid of the foregoing means. We know that in pleurisy and peripneumony, and in other inflammations, the above means render blistering highly efficacious; and that, in acute rheumatism, it is a speedy and effectual assistant in removing the complaint:—why then may it not have a good effect in promoting resolution in incipient cancers as well as in inflammation? As in the one case suppuration is prevented, so in the other may the progress of cancer be arrested; and its tendency to increase, with pain from distention and alteration taking place within the gland, be prevented.*

"From the irritability of most cancers, some practitioners have relied much on the effects of nature, and have considered that almost all topical and general modes of treatment have rather exasperated than retarded the progress of the disease. If this observation be confined to the use of most external and all internal *stimulants*, there is I believe much truth in the doctrine; but experience has brought me to think that a treatment both local and general, of a *sedative* kind, is of singular service.

"First, With respect to repellents, externally, such are the preparations of Lead, which have in all ages been recommended, in some form or other, as palliatives in this disease, much might be said, upon the authority of ancient as well as modern writers. Not however to dwell on the assertions of others, but merely to give the result of my experience, I have found some of them useful, in the early state of the disease, before ulceration, both as sedatives and defensives. The latter may appear to be an obsolete and antiquated term, but it is of some consequence in an incipient scirrhus or cancer, to defend it from external injury, and from friction, and from being involuntarily scratched. This may be done by the wearing of a thin coat of good Emplastrum Simplex, spread on soft leather:—Nutritum also has been used on the most irritable of all sores, viz. those arising from

* "If a blister be thought advisable, two circumstances should be attended to. The skin which covers the tumor should be in an uninflamed state, and before it takes on the shining purplish hue of cancer, when the excoriations will be likely to heal,—in which case it may be repeatedly renewed;—but, if applied when in an irritable and highly-inflamed state, I suspect it would tend to ulcerate the part, and bring on, prematurely, the cancerous mischief. And, moreover, before its application, evacuations, particularly that of blood-letting by leeches, should be used; and, as these make small irregular wounds, I have never applied them to the skin which covers the tumor, even when that skin has been without the purplish blush, but to such parts surrounding the tumor on which there is neither irritation nor inflammation,—and here they may be applied with safety.

"As a general caution, this is worth attending to, for I am certain I have seen ulceration greatly accelerated, when leeches have been suffered to draw blood from the surface of an irritable and inflamed cancer.

burns; burns; and it is a soft and easy application, if the ingredients of which it is composed are good, without any portion of rancid oil. Unguentum Tripharmacum; Aq. Litharg. Acetati; Solutio Salis Ammoniaci, *in Aquâ*; have had extremely good effects.

"A very intimate friend of mine, an eminent surgeon, was much tormented with the shingles, and he applied to me, for, what he was pleased to call, *my* plaister: he used it, and not only experienced instant relief, but a speedy cure. The history of the plaister is this:—A lady of my acquaintance having learned that a female had a certain *nostrum* for the cure of cancer, which she kept secret, by dint of persuasion induced her to give up the Recipe; and this it is which follows in the note below*. I am of the opinion of Lord Bacon, that every Recipe should be given faithfully, and I therefore communicate it as I received it:—the medical reader may make the composition of any degree of consistence he may think proper.

"The uses to which such a topic may be applied are many. Most of our plaisters, and many of our ointments, are heating and strong stimulants; but this is a sedative, and a very valuable one.

"When preparations of lead are employed as sedatives, there can be no objection to an admixture of Opium: it may be combined various ways, with this and other sedatives. If by such combinations, relief from pain and irritation, and a retardation of the progress of the disease, be effected, scirrhus may become stationary. There is reason for believing that opium applied externally to a scirrhus, though the skin be whole and without ulceration, will exhibit its sedative qualities.

"It has been stated that the glands take on, at certain periods of life, an altered state, and, in consequence, new actions and a new disposition;—that erysipelas, gout, and a variety of local affections, such

---

* "Put a complete handful of the inner bark of green elder into a quart of the best olive-oil, and let it soak for two or three days. Then set it over a gentle fire, in a brass skillet, and let it stew until the elder-bark be crisp. Strain it off, and add more oil, to make up a full quart, and set it aside to cool. To this quart put half a pound of red lead, and half a pound of white lead, both finely powdered and sifted. Boil them *gently* till the mass comes to a salve, keeping it stirring the whole while with a stick of elder. When it is done, and going to be cooled, stir into it two drachms of powdered camphor.—(It will take probably two or three hours boiling.)

"To know when it is enough, drop some of it on a plate, and when it is tenacious, without sticking to the fingers, it will do. Have ready as much old linen-cloth, quite clean and free from starch or hems, as will soak the whole up; to be put in whilst the salve is liquid. Take it out; hang it, as smooth as you can, over lines, and let it dry without sun or air:—then rub it on marble with a slick-stone until quite smooth; and keep it rolled in paper for use.—It may be made in brass, bell-metal, silver, or earthen-ware; the first is best.—March or September are the right months for making it; and the longer it is kept, the better it gets.—N.B. A very skilful hand may spread it on one side of the linen-cloth, if the accumulation of plaister on both sides, by dipping it, be found inconvenient.

"It is good for all outward complaints, in man or beast. For a strain, you should cover the whole ancle with leather or paper, of the size of the plaister, and over it; and let it remain on as long as it will stick, viz. a month, or more. For a wound that discharges, lift up the plaister, wipe it and the wound, and down with it again; and change it only when absolutely necessary: perhaps, once in two days, or oftener."

as as asthma, sore legs, and an habitual disposition to chronic diseases, are incident to late periods of life, at which time scirrhous tumors become generally troublesome, and the cancerous tendency shows itself. If at such periods there exist this kind of diathesis, especially in females, how easy may the transition be to the breast or uterus, causing local mischief there, in lieu of other more usual affections!—I am convinced that many defluxions on glandular parts have thus originated.

"With respect to relief under such circumstances, I apprehend that a vast deal must depend upon regimen and diet, and on evacuations, particularly bleeding and purging. As to the first, the French seem to me to have carried this point, at the critical period of the cessation of the catamenia, much further than we have; and as a greater degree of plethora then probably prevails, the practice appears well founded. I fear, with regard to this evacuation, we are too remiss.

"An abstemious diet is also of the utmost importance; and of equal necessity is—due attention to the state of the bowels. Without these, very little progress can be made in our attempts to keep a scirrhous tumor quiet, or prevent its speedy progress towards a cancerous state. The same practice which the late Dr. Russel found necessary of purging thoroughly with sea-water, and thereby emptying the intestinal canal, and setting the absorbent lacteals at liberty, in obstructions of the mesenteric glands, and in scrofulous tumors, would be of great use in all scirrhi and in all tumors having a cancerous tendency.

"It was long ago remarked, I think by the judicious Mr. Samuel Sharp, that a milk and vegetable diet, with the use of purging salts, would often tend to keep a scirrhous affection quiet. How much more likely are the same means to give relief *now*, when, in addition, it has been discovered that LEECHES may be applied to the neighbourhood of the diseased part, without irritating the tumor; a practice which was not prevalent in *his* time! Nor was the immersion of the whole body in TEPID SEA or WARM WATER, at that time conceived to have any salutary effect:—nor was the *local* effect of SEA-WATER or SEA-WRACK, (alga marina,) so well ascertained as it is now.

"With respect to *leeches*, I have known more than one hundred used within the space of a few months, with singular benefit; and, if the orifices from a few leeches be kept bleeding, for some time after their commencement, by means of a sponge and warm water, the evacuation is by no means striking,—for it is powerfully sedative.

"I do not pretend to say what will *cure* a scirrhus or a cancer without the knife, (and even that is very far from being infallible,) but this I know—that *sedatives*, when applied to the part, or to its neighbourhood, or to the system in general, will frequently check its progress for a long time; and I also know that whatever applications are used locally or generally to the system of an inflammatory and heating nature, accelerate and increase the violence of the symptoms."

If in this posthumous work the author had bequeathed a cure for *Carcinoma*, most valuable indeed would have been the legacy: but, though we have not received this from him, we cannot but allow that many practical remarks of importance, and methods of treating the disease, fairly resulting from observation, and therefore highly deserving regard, have been preserved to society by the care of DR. GOWER.

